# Odonata Assemblages in Urban Semi-Natural Wetlands

**DOI:** 10.3390/insects15030207

**Published:** 2024-03-20

**Authors:** Marina Vilenica, Andreja Brigić, Ana Štih Koren, Toni Koren, Mirela Sertić Perić, Bruno Schmidt, Tomislava Bužan, Sanja Gottstein

**Affiliations:** 1 Faculty of Teacher Education, University of Zagreb, Trg Matice Hrvatske 12, 44250 Petrinja, Croatia; 2 Department of Biology, Division of Zoology, Faculty of Science, University of Zagreb, Horvatovac 102a, 10000 Zagreb, Croatia; andreja.brigic@biol.pmf.hr (A.B.); mirela.sertic.peric@biol.pmf.hr (M.S.P.); sanja.gottstein@biol.pmf.hr (S.G.); 3 Association Hyla, Lipovac I 7, 10000 Zagreb, Croatia; ana.stih@hhdhyla.hr (A.Š.K.); toni.koren@hhdhyla.hr (T.K.); bruno.schmidt@hhdhyla.hr (B.S.); 4 Department of Biology, Josip Juraj Strossmayer University of Osijek, Trg Svetog Trojstva 3, 31000 Osijek, Croatia; tomislava.buzan@gmail.com

**Keywords:** man-made habitats, large rivers, oxbow lakes, life history traits, dragonflies, damselflies

## Abstract

**Simple Summary:**

Aquatic habitats in urban areas are often important for conservation of local biodiversity. Although Odonata are widely used as ecological indicators of freshwater habitat integrity and health, our understanding and knowledge of their ecological requirements in urban landscapes is not yet complete. Therefore, the taxonomic and functional diversity of Odonata assemblages was analyzed in a semi-natural wetland in the Croatian capital in the summers of 2020 and 2023. The two main habitat types, anthropogenically disturbed and natural oxbow lakes, mostly had comparable assemblage metrics. However, we found significant differences in relation to the time scale, where most metrics were lower in 2023, indicating the negative impact of extreme climate events (including droughts) that occurred in this region after 2020. As even some species of conservation concern were detected, our results indicate that semi-natural urban wetlands, especially natural oxbow lakes, have great potential to function as good habitats for Odonata.

**Abstract:**

As the human population in urban areas is continuously growing, urbanization is one of the greatest threats to biodiversity. To mitigate the negative effects, the inclusion of blue zones (aquatic habitats) in modern urban development practices is strongly recommended, as they could be beneficial for the local biodiversity conservation. Odonata are a flagship group and are widely used in freshwater conservation as ecological indicators of habitat integrity and health. However, our understanding of their ecological requirements in urban landscapes is not yet complete. Therefore, we analyzed the taxonomic and functional diversity of Odonata in a semi-natural wetland in the Croatian capital. This study was conducted in the summers of 2020 and 2023. Most taxonomic and functional assemblage metrics were comparable between the two main habitat types, anthropogenically disturbed and natural oxbow lakes. However, significant differences were found in relation to the time scale, where most metrics were lower in 2023, indicating the negative impact of extreme climate events (including droughts) that occurred in this region after 2020. With 19 species recorded, our results indicate that semi-natural urban wetlands, especially natural oxbow lakes, have great potential to function as good habitats for Odonata, where even some species of conservation concern were detected. When developing landscape management plans in urban areas, it is essential to consider the importance of habitat heterogeneity in terms of good structure of aquatic macrophytes (presence of submerged, emergent and floating vegetation), which would ensure the most suitable habitat conditions for local Odonata species.

## 1. Introduction

Currently, more than half of the world’s population resides in urban areas [1], and this number is expected to increase by a further 10 percent by the beginning of the next decade [2]. Urbanization is the second largest cause of natural habitat destruction worldwide [3] and, as a form of land-use change that leads to habitat fragmentation and isolation, is one of the greatest threats to biodiversity [4]. Therefore, the inclusion of green (vegetation) and blue zones (aquatic habitats, such as streams, rivers, canals, wetlands, ponds, lakes and reservoirs) in urban design is strongly encouraged in modern urban development practice as they offer potential benefits for local biodiversity conservation and improve local microclimate conditions [5]. However, despite their potential conservation value, the environmental conditions in urban habitats are far from optimal for most organisms.

In urban environments, both terrestrial and aquatic habitats undergo significant changes characterized by increased local minimum temperatures compared to their natural counterparts, contributing to the urban heat island (UHI) effect [6]. These changes in abiotic conditions are primarily due to shifts in vegetation cover, including the transition from natural vegetation to non-native species or the complete removal of vegetation. Such changes can amplify the UHI effect by influencing surface albedo and heat absorption [7]. In addition, changes in vegetation and abiotic conditions can lead to excessive habitat fragmentation and thus degradation of natural landscapes [1,8]. These changes have cascading effects on abiotic conditions throughout the urban ecosystem also affecting urban freshwater habitats. For example, urban aquatic habitats might demonstrate elevated temperatures due to the UHI effect, changes in composition of aquatic macrophytes from non-native vegetation, fragmented ecosystems caused by urban development, straightened river channels altering flow patterns, and reduced water levels resulting from water abstraction practices [9]. A comprehensive understanding of the complex relationship between vegetation changes and their effects on abiotic conditions such as temperature and hydrology in urban green and blue zones is crucial for effective urban planning and conservation strategies [1,6,8,9].

In addition, urban freshwater habitats are often exposed to various sources of pollution [10,11]. Eutrophication and phytoplankton blooms (including toxic cyanobacteria) are common in urban standing waters due to high nutrient loading (phosphorus and nitrogen) from stormwater and urban runoff (e.g., municipal wastewater discharge, sewage treatment plants and sewage overflow) [11,12], resulting in high oxygen consumption in the sediment and a reduction of light in the water column, which limits the growth of aquatic macrophytes, leading to a shift in their communities or their complete disappearance [13]. Moreover, water and sediment in urban freshwater habitats often contain high levels of heavy metals and toxic organic compounds originating from traffic, households, industry, fertilizers and chemicals used to control unwanted organisms (e.g., mosquitoes) or to improve water clarity [5].

Anthropogenic pressure in urban habitats has a synergistic effect on benthic organisms, leading to the disappearance of sensitive species and a decline in the fitness of those that remain [5,13,14]. This process leads to a biotic homogenization of benthic communities and increases the risk of local extirpation [1,15,16]. However, studies have shown that different taxonomic groups of aquatic macroinvertebrates respond differently to urban biodiversity drivers (e.g., habitat size, margin naturalness, water temperature, pollution level, aquatic and riparian vegetation, presence of fish or invasive species) [5]. Furthermore, it was shown that urban ponds typically harbor less-diverse macroinvertebrate assemblages compared to their non-urban counterparts [6,17,18,19]. These assemblages predominantly comprise widely distributed eurytopic taxa characterized by high tolerance to environmental conditions and robust dispersal abilities [20,21]. Some generalist taxa, such as Oligochaeta and Chironomidae, can even thrive in such habitats and their assemblages can be abundant and species-rich [22,23].

If urban lentic waters are properly managed, they could play an important role in maintaining freshwater biodiversity and even provide a habitat for some endangered species [24,25]. However, if they are polluted and not properly constructed and managed, some of them could represent low-quality habitats where species cannot complete their life cycle. Such habitats then act as ecological traps, i.e., habitats that are mistakenly considered by organisms to be more suitable than habitats with better environmental conditions, increasing the risk of local extinction of some populations [19,26].

In freshwater conservation, aquatic insects of the order Odonata are a flagship group, often used as ecological indicators of habitat integrity and condition [27,28]. They are widely distributed, occupy an important position as predators in aquatic and terrestrial food webs, exhibit relatively high taxonomic diversity with different species requiring different environmental conditions, and have short but complex life cycles that cross the aquatic–terrestrial interface and that allow them to respond quickly to changes in both aquatic and adjacent terrestrial habitats [29,30,31]. Due to these characteristics, Odonata are sensitive indicators of freshwater habitat change and anthropogenic impacts [32,33]. Despite the growing number of studies on Odonata in urban habitats [25,34,35,36], our understanding of their ecological requirements in urban wetlands in large cities is not yet complete.

Therefore, the main aims of this study were: (i) to determine the taxonomic and functional diversity of Odonata in two habitat types—anthropogenically disturbed and natural oxbow lakes—in an urban wetland complex, (ii) to detect temporal changes in those metrics between the two sampling years and (iii) to assess the conservation value of the two habitat types.

## 2. Materials and Methods

### 2.1. Study Area

The study area is located in the southeastern part of the city of Zagreb, Croatia, about 4 km from the city center [37]. The Savica Lakes are a complex of semi-natural wetlands along the Sava River. The complex includes the old oxbow lakes of the Sava and several abandoned gravel pits created by the expansion of the Sava oxbow for the purpose of gravel extraction. In 1964, an embankment was constructed between the lake complex and the Sava River, separating the wetland from the river [38]. Since the lakes are hydraulically connected to the Sava River, the water level in the lakes depends on the water level of the Sava River [38].

The Savica area has a total water surface of 30 ha and is divided into two parts [37]. The upper lakes are located north of the old, disused railroad line, which was used in the past to supply coal to the local thermal power plant. The upper lakes are connected to the lower lakes located south of the railway by drainage pipes [38].

The man-made gravel paths surrounding the lakes are on both sides overgrown with trees (predominantly *Salix alba* L., *Populus nigra* L., *Populus alba* L., *Alnus glutinosa* (L.) Gaertner, *Robinia pseudoacacia* L.) and shrubs (such as *Rubus* spp., *Clematis vitalba* L.) [37]. The aquatic vegetation consists mainly of emergent and floating macrophytes (such as *Myriophyllum spicatum* L., *Nuphar lutea* (L.) Sm. in Sibith. et Sm., *Ceratophyllum demersum* L.), and the most widespread is *Myriophyllo*-*Nupharetum luteae* (W. Koch 1926) Hueck 1931 association. In the shallow parts of the lakes, close to the shoreline, *Lemno-Spirodeletum polyrhizae* W. Koch 1954 and *Nymphoidetum peltatae* Bellot 1951 associations are developed. The riparian vegetation is generally well developed and uniform on all lakes, and is represented by the community of periodically flooded banks (*Nanociperion* W. Koch 1926) (including *Cyperus* sp., *Carex* sp., *Juncus* sp.). Additionally, some patches of the lakes’ banks are covered with *Phragmitetum australis* (Gams 1927) Schmale 1939 and *Typhetum latifoliae* (Soó 1927) Now. 1930 associations [37].

The great importance of this area lies in the fact that it is the only relatively well-preserved wetland within the city of Zagreb, which is a refuge for many vertebrate and invertebrate species [39]. Therefore, the area has been protected as a significant landscape since 1991 due to its high biological value (e.g., 174 bird species and 16 mammal species) [37,40]. Nowadays, the old oxbow lakes and gravel pits are mainly used for fishing purposes [37].

However, some of the lakes are anthropogenically influenced in several ways. Firstly, the cooling water from the Zagreb thermal power plant is discharged directly into the upper lakes. Although the upper and lower lakes are connected, there is a temperature difference between them [38]. Due to the inflow of warm water from the power plant, the upper lakes sometimes do not freeze over in the winter months, while the other lakes regularly freeze over every year. Furthermore, the densely populated suburb of Šanci is located near the Savica Lakes, while a large part of the area is also surrounded by agricultural land with greenhouses.

### 2.2. Study Sites and Odonata Sampling

**Figure 1:**
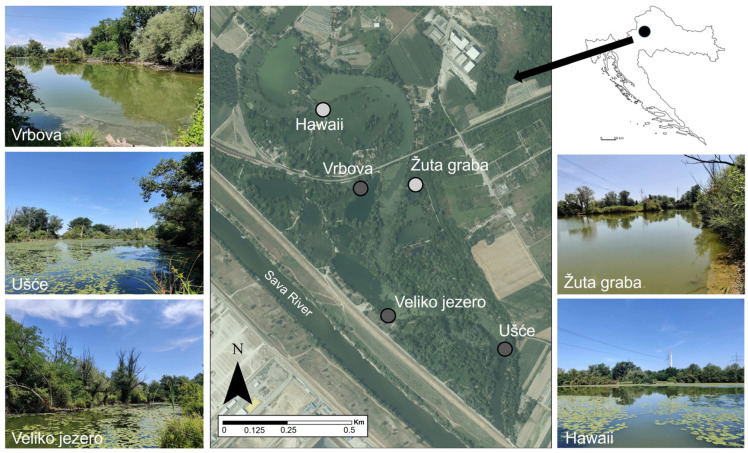
Geographical depiction of the Savica significant landscape in the city of Zagreb, Croatia, featuring photographic examples of the study sites. Legend: light grey spots—anthropogenically disturbed lakes, dark grey spots—natural oxbow lakes.

Our study included a total of five lakes within the Savica urban lakescape, belonging to two habitat types that can be distinguished based on their habitat morphology: anthropogenically disturbed lakes (Hawaii and Žuta graba) (abandoned gravel pits) and natural oxbow lakes (Vrbova, Veliko jezero and Ušće) (remnants of the Sava River oxbow) (Figure 1).

Adult Odonata were investigated at each study site in the summer months of 2020 and 2023, with six sampling occasions between June and September. At each lake, Odonata species were investigated for a period of 60 min (until no additional species were detected) along a transect following the lake’s shoreline. All adults observed within ≈5 m of the transect route were documented, identified and numbers of each species were counted (high abundances of damselflies, if present, were immediately estimated). Fieldwork was conducted on sunny days, between 9 a.m. and 4 p.m. Adults were mostly observed visually and identified by eye or binoculars with close vision. Some species were sampled using an entomological net (e.g., the species of the genus *Sympetrum*) and released after identification.

### 2.3. Environmental Variables

During three sampling occasions in 2023, the following environmental variables were measured in triplicates at each study site: water temperature, dissolved oxygen saturation (using the oximeter OXI 96, WTW GmbH, Weilheim, Germany), conductivity (using the conductometer Sension 5, Hach, Loveland, CO, USA), and pH (using the pH-meter 330i, WTW GmbH, Weilheim, Germany). In addition, 1 L water samples for laboratory analysis of the water (total water hardness, chemical oxygen demand, nitrite and nitrate concentrations) were taken from the same locations at each study site. In the subsequent laboratory analysis, total water hardness and nitrite and nitrate concentrations were measured according to the standard procedures [41], while the assessment of chemical oxygen demand, which serves as a surrogate parameter for dissolved organic matter in water, was carried out based on the methods described by the Deutsches Institut für Normung [42], as described in Sertić Perić et al. [43].

### 2.4. Data Analysis

To assess the taxonomic diversity of Odonata, Odonata assemblage metrics (species richness (S), abundance (N), Shannon (H′) and Simpson diversity indices (1 − λ)) were calculated for each sampling event at each study site in the two habitat types (anthropogenically disturbed and natural oxbow lakes).

To assess the similarities of Odonata assemblages between the two habitat types, a cluster analysis based on the Bray–Curtis similarity matrix was performed. Species abundance data were log (x + 1) transformed prior to analysis. A SIMPER (similarity percentage) analysis was performed to assess the species predominantly responsible for the similarities between sites of the same habitat type. SIMPER analysis was based on the log-transformed species data and preformed using the Bray–Curtis similarity matrix. Odonata assemblage metrics, Bray–Curtis similarity index, cluster and SIMPER analyses were performed using the PRIMER 6.0 software package [44].

Prior to further analyses, the normality of the taxonomic and functional assemblage metrics as well as the physico-chemical parameters was tested using the Shapiro–Wilk test in SPSS Statistics ver. 27.0 [45].

To assess the conservation value of the habitat types studied, the Dragonfly Biotic Index (DBI) was calculated, an index commonly used to assess the ecological integrity and health of freshwater ecosystems using Odonata assemblages [46]. This index weights species according to their geographic distribution, conservation status and sensitivity to anthropogenic habitat disturbance. The following subcategories were used in the analysis: national distribution (according to [47]), national red-list classification (according to [48]), and species sensitivity to habitat change (assessed based on expert knowledge). Each subcategory can be scored from 0 to 3 points (i.e., a widespread, non-threatened species that is highly tolerant to anthropogenic disturbance receives 0 points (0 + 0 + 0), while a species with a highly restricted distribution that is also highly threatened and extremely sensitive to habitat disturbance receives 9 points (3 + 3 + 3)). For each habitat type, a standardized DBI score was calculated by summing the DBI scores of all species occurring in a given habitat type and dividing by the number of species occurring in that habitat type, treating sampling events as replicates.

To quantify the functional diversity of Odonata assemblages in two main habitat types, the Rao quadratic diversity (RaoQ) coefficient was used, which is a measure of the convergence or divergence of traits compared to random expectations [49]. A total of 20 functional traits from five trait groups were used to calculate RaoQ coefficient (taken from [50,51]): (i) body shape (Zygoptera and Anisoptera), (ii) preference for lateral connectivity: (a) eupotamon = main channel and connected lateral arms; (b) parapotamon = lateral arms connected only at the downstream end at mean water level; (c) plesiopotamon = no connectivity with the main channel at mean water level, including lakes where macrophyte coverage does not exceed 20%; (d) palaeopotamon = no connectivity with the main channel at mean water level, including lakes and pools, where coverage by macrophytes exceeds 20%; (e) temporary water bodies = temporary pools where the water level depends primarily on ground water levels, (iii) current preference: (a) limnophilous = mostly occurring in stagnant waters, rarely also in slow-flowing lotic habitats; (b) limno- to rheophilous = prefer stagnant waters, but often also occur in slow-flowing lotic habitats; (c) rheo- to limnophilous = prefer slow-flowing streams and their lentic zones; (d) rheophilous = occur in lotic habitats, preferably with moderate and fast flow velocity, (iv) dispersal capacity (high, medium) and (v) reproduction (reproduction mode and the form and location of oviposit clutches): eggs laid: (a) attached to substrate, (b) in the substrate, (c) not attached to/in substrate, (d) in open water, (e) into plant tissue, (f) on plant material, (g) on exposed soil or rock. For each functional trait in Odonata assemblages, the community weighted means (CWMs) were calculated to quantify shifts in mean trait values within assemblages, resulting from environmental selection for specific functional trait categories [49] using the CANOCO package version 5.15 [52].

A series of generalized linear mixed models (GLMMs) were constructed to assess differences in physico-chemical water properties, taxonomic and functional Odonata assemblage metrics, and DBI between the two habitat types. In all constructed models, sites (level 1) nested within the habitat type (level 2) were included with sampling events as repeated measures (year, month). The interaction effect between habitat type and year was used as a fixed effect in all models. To account for variation caused by potential differences among study sites and sampling events, sites and sampling events (months) were included in all models as random effects, with first-order autoregressive (AR1) covariance type, which was assumed for repeated measures over time [53]. GLMMs with normal distribution and with log link function were constructed for targeted variables: species richness, abundance, Shannon diversity and RaoQ, while gamma distribution with log link function was used for other non-normally distributed variables. Estimation was built by Relative Hessian Convergence. Degrees of freedom were computed for significance test using Satterthwaite approximation that is used for small sample size and unbalanced data. Pairwise contrasts of estimated means between habitat types (anthropogenically disturbed vs. natural) and years (2020 vs. 2023) of estimated means were applied using a least significant difference (LSD) *post-hoc* test. The above analyses were performed using SPSS Statistics ver. 27.0 [45].

## 3. Results

### 3.1. Environmental Variables

Anthropogenically disturbed and natural oxbow lakes differed significantly in terms of oxygen saturation and nitrite concentration in the water (Table 1). Anthropogenically disturbed habitats had significantly higher oxygen saturation and nitrite concentration compared to the natural oxbow lakes (Table 1). Other measured water parameters did not differ significantly between the two habitat types (Table 1).

### 3.2. Odonata Species and Their Threat Level

A total of 19 species were recorded in two habitat types in the studied urban wetland in Croatia (Table 2). At the anthropogenically disturbed lakes, 13 species were recorded, while all 19 species were detected at the natural oxbow lakes (Table 2). The SIMPER group similarity analysis (Table 3) showed that *Ischnura elegans* (Vander Linden, 1820) and *Platycnemis pennipes* (Pallas, 1771) were the dominant species at both habitat types (Table 3). *Aeshna isoceles* (Müller, 1767) and *Lestes sponsa* (Hansemann, 1823) are listed as near threatened species (NT) in the Croatian Red List of Odonata [48].

**Figure 2:**
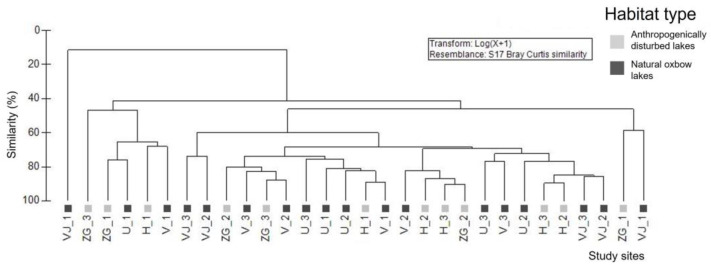
Cluster analysis of study sites belonging to anthropogenically disturbed and natural oxbow lakes in the Savica urban lakescape in the city of Zagreb, Croatia, based on the composition of Odonata assemblages. Legend: H—Hawaii, ZG—Žuta graba, VJ—Veliko jezero, V—Vrbova, U—Ušće lakes. Numbers 1–3 are sampling events.

The cluster analysis revealed a significant degree of similarity (ca. 40%) among the study sites associated with the two distinct habitat types. This similarity was identified through an examination of their Odonata assemblages, highlighting the spatial extent of these assemblages within the Savica urban lakescape (Figure 2).

### 3.3. Odonata Assemblages and Their Conservation Value

**Figure 3:**
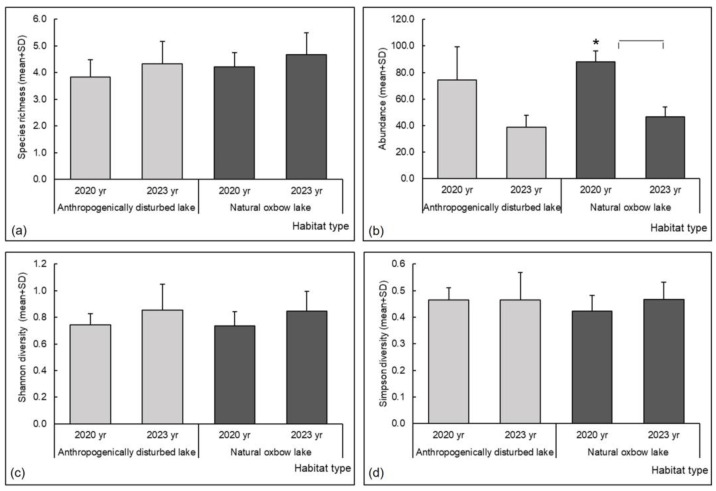
Odonata taxonomic assemblage metrics at anthropogenically disturbed and natural oxbow lakes in the Savica urban lakescape in the city of Zagreb, Croatia: (**a**) species richness, (**b**) abundance, (**c**) Shannon diversity index, (**d**) Simpson diversity index. Asterisks indicate significant differences in metrics between the different years of the study (* = *p* < 0.05).

Among the taxonomic parameters, only abundance differed significantly in relation to the time scale at natural oxbow lakes, and it was higher in 2020 compared to 2023 (Table 4, Figure 3b). Odonata species richness, Simpson and Shannon diversity indices were comparable between the two habitat types and regarding the time scale (Table 4, Figure 3a,c,d).

**Figure 4:**
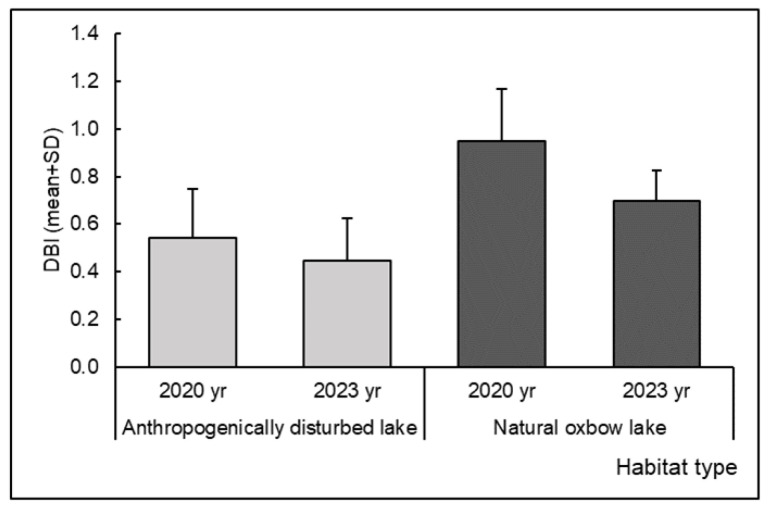
Dragonfly Biotic Index (DBI) calculated for anthropogenically disturbed and natural oxbow lakes in the Savica urban lakescape in the city of Zagreb, Croatia.

No significant differences were found in DBI values between the habitat types, nor regarding the time scale of the study (Table 4, Figure 4).

### 3.4. Functional Diversity of Odonata Assemblages

**Figure 5:**
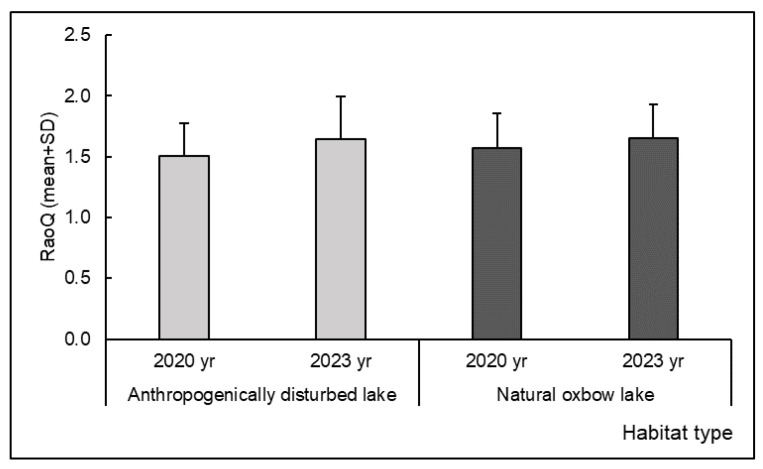
Functional diversity (RaoQ index) of Odonata assemblages at anthropogenically disturbed and natural oxbow lakes in the Savica urban lakescape in the city of Zagreb, Croatia, presented as mean and standard deviation (SD).

**Figure 6:**
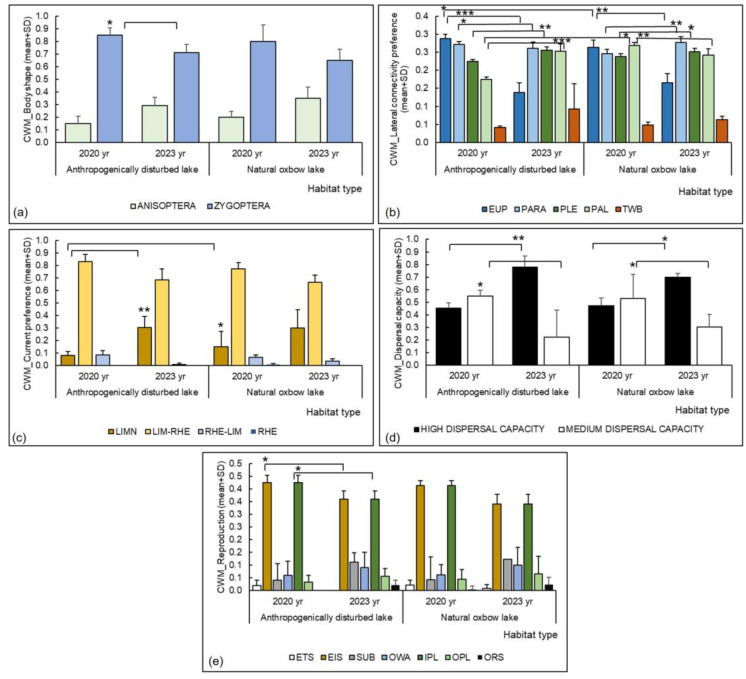
Functional traits of Odonata at anthropogenically disturbed and natural oxbow lakes in the Savica urban lakescape in the city of Zagreb, Croatia, presented as mean and standard deviation (SD) in relation to: (**a**) body shape, (**b**) habitat preference of species for lateral connectivity, (**c**) current preference of species, (**d**) species dispersal capacity, (**e**) reproduction type. Asterisks indicates a significant difference between the habitat types and between the different years of the study (*** = *p* < 0.001, ** = *p* < 0.01, * = *p* < 0.05). Legend: yr—year; CWM—community weighted mean; EUP—eupotamon, PARA—parapotamon, PLE—pleisiopotamon, PAL—palaeopotamon, TWB—temporary waterbodies; LIMN—limnophilous, LIM-RHE—limno- to rheophilous, RHE-LIM—rheo- to limnophilous, RHE—rheophilous; ETS—eggs laid attached to substrate, EIS—eggs laid into the substrate, SUB—eggs laid not attached to or in substrate, OWA—eggs laid in open water, IPL—eggs laid inside plant tissue, OPL—eggs laid onto plant material, ORS—eggs laid on exposed soil or rock.

The functional diversity (RaoQ) of Odonata assemblages was comparable between anthropogenically disturbed and natural oxbow lakes (Table 5, Figure 5). However, significant differences were found for several functional traits (Table 5, Figure 6).

In terms of lateral connectivity preference, eupotamon element was significantly different between habitat types in 2020 (higher in anthropogenically disturbed lakes) (Table 5, Figure 6b). Significant differences were found at both habitat types with respect to the time scale (higher in 2020) (Table 5, Figure 6b). Significant differences in parapotamon and plesiopotamon were observed at both habitat types with respect to time scale (parapotamon was higher at anthropogenically disturbed habitats in 2020, at natural oxbow lakes in 2023; plesiopotamon was higher at both habitat types in 2023) (Table 5, Figure 6b). A significantly higher proportion of palaeopotamon was found at natural oxbow lakes in 2020 compared to anthropogenically disturbed lakes (Table 5, Figure 6b). Differences were also observed regarding the time scale at both habitat types (higher at anthropogenically disturbed habitats in 2023 and at natural oxbow lakes in 2020) (Table 5, Figure 6b).

In terms of current preference, a higher proportion of limnophilous species was found at natural oxbow lakes than at anthropogenically disturbed oxbows in 2020 (Table 5, Figure 6c). Significant differences were observed regarding the time scale at anthropogenically disturbed lakes (higher in 2023) (Table 5, Figure 6c).

Regarding the dispersal ability, differences were observed on the time scale at both habitat types (higher proportion of species with high dispersal ability in 2023, higher proportion of species with medium dispersal ability in 2020) (Table 5, Figure 6d).

## 4. Discussion

### 4.1. Blue Zones in a City Have Great Potential to Function as Good Habitats for Odonata, but Time Combined with Climate Extremes Affects Their Functional Trait Representation

Most physico-chemical water parameters were comparable between the anthropogenically disturbed and natural oxbow lakes; however, significant differences were found in the concentration of nitrites and water oxygen saturation. Waterbodies located in urban areas generally have elevated nutrient concentrations (nitrites, nitrates, phosphates) originating from sewage discharge, wastewater from industrial plants, city parks and/or sporadic agricultural areas where various pesticides and fertilizers are used for growing and maintaining plants. Also, rainwater runoff often brings with it drainage water from the surrounding city roads [10,13,16]. Although anthropogenically disturbed lakes in the studied urban lakescape had significantly higher concentrations of nitrites in water compared to natural oxbow lakes, those concentrations were still very low considering the permitted limit values in surface waters (maximum 1 mg/L) [54].

The interplay between water temperature and oxygen saturation emphasized by Wetzl and Likens [55] proved to be a central factor influencing the concentration of dissolved oxygen in aquatic ecosystems. Our observations showed that natural oxbow lakes had, on average, lower oxygen saturation than anthropogenically disturbed lakes. In general, warmer waters have higher oxygen saturation, but, paradoxically, dissolved oxygen levels decrease with increasing water temperature [55]. In the summer months, particularly in the warmer upper layer of the lake where our physico-chemical measurements were conducted, total oxygen availability may be limited by temperature. This limitation exists even when the water is 100% saturated with oxygen, which can result in suboptimal dissolved oxygen levels for various organisms—a scenario that is likely to occur in the Savica Lakes. The influence of aquatic plants on oxygen saturation further adds to the complexity of this relationship. The process of photosynthesis by aquatic plants increases the oxygen content in the water [55]. In particular, lakes with high oxygen saturation, such as Hawaii Lake with its reed beds and Vrbova and Žuta Graba lakes with their submerged aquatic vegetation and water lilies, had a higher proportion of aquatic plants compared to lakes with lower oxygen levels. This observation underlines the role of aquatic vegetation in increasing oxygen saturation and thus in shaping oxygen dynamics in these freshwater ecosystems.

Our results indicate the importance of blue zones in large cities as habitats for Odonata. As this study shows, the presence of natural habitat features (habitat morphology and vegetation structure) is beneficial for many Odonata species and should be considered in landscape management plans in urban areas to ensure important habitat complexity for aquatic insects such as Odonata [34,56]. Due to the mutual proximity of the study sites, similar values of physico-chemical water properties, generally well-developed aquatic vegetation [37] and the high dispersal mobility of adult Odonata (which allows the possibility that many of the individuals observed at a particular lake emerged from another lake) [57], most of taxonomic assemblage metrics were comparable between the anthropogenically disturbed and natural oxbow lakes, similar to the results of Dolný et al. [58].

However, we observed differences in Odonata abundance regarding the time scale: it was significantly higher at natural oxbow lakes in 2020, which might be related to the rather harsh climate conditions in recent years. From the winter of 2021, much of Europe was affected by widespread drought. The extreme precipitation deficit led to a decline in surface and groundwater levels, resulting in many perennial aquatic habitats becoming intermittent, negatively impacting both aquatic and terrestrial ecosystems [59]. Climate change also led to intense summer thunderstorms, which were particularly strong in the studied region in 2023 [60]. Given the severe drought in 2022 and the violent storms with high rainfall and strong winds in spring and summer 2023, it is conceivable that the survival of numerous Odonata individuals—both eggs and nymphs in 2022 and adults in 2023—was affected. This is consistent with the results of relevant studies (e.g., [61,62]), which similarly emphasized ecological disturbances in Odonata populations in response to comparable climatic stressors.

Similar to most taxonomic metrics, functional diversity of Odonata assemblages was found to be comparable between the two habitat types, indicating that species occupying these habitats have similar traits that allow their success in urban areas. Our results differ from previous studies that found a decline in Odonata functional diversity with increasing anthropogenic pressure [32,63]. These discrepancies could be due to complex interactions influenced by (i) historical and geological factors, (ii) similar (micro) habitat conditions in the studied area and (iii) the presence of hydrological stressors. For example, the shared geological features of the two habitat types can provide comparable ecological niches for Odonata species, but also habitat conditions such as vegetation composition and water quality may contribute to maintaining functional diversity despite anthropogenic pressure (cf. [64,65]). As pointed out by Willigalla & Fartmann [64] and the references herein, a considerable number of Central European cities (e.g., Zagreb) are located in floodplains adjacent to large rivers and in regions characterized by pronounced biological and geological diversity. Therefore, these areas already had a considerable habitat and species richness even before human settlement. It can therefore be assumed that cities in such locations naturally have a high abundance and diversity of Odonata, which could also explain our results independent of anthropogenic influence. Moreover, hydrological stressors such as altered water or nutrient levels can impact Odonata assemblages and their functional diversity (cf. [65,66]). The urban lakescape of Savica likely exhibits a stormpond effect, similar to the findings of Holtmann et al. [66], who observed that urban stormwater ponds, characterized by a warmer microclimates and relatively low nutrient concentrations (as in lakes included in this study), harbor richer and more diverse Odonata assemblages, including threatened species. The absence of a noticeable decline in functional diversity can therefore be attributed to the mitigating effect of the Sava oxbow lakes on the studied habitats. Moreover, it is likely that habitat diversity in terms of different lateral habitat connectivity, which as an environmental factor largely depends on hydrological conditions, largely influences the spatial and temporal patterns of taxonomic and functional diversity of Odonata.

In our study, the structure of Odonata assemblages differed with respect to some life history traits, especially in terms of time scale. Both habitat types were predominantly inhabited by generalists found in different types of freshwater habitats [51]. Although significant differences were found in terms of species preference for lateral habitat connectivity and flow, the pattern was not clear for either habitat type. However, these traits and their differences with respect to time scale could be related to the dispersal ability trait. The stronger representation of the trait “high dispersal ability” in 2023 and the trait “medium dispersal ability” in 2020 could also be a consequence of the harsh climatic conditions that occurred in the time frame of this study, which favored the persistence of generalist lentic species with high dispersal ability (such as *Crocothemis erythraea* (Brullé, 1832), *Orthetrum albistylum* (Selys, 1848), *Anax imperator* Leach, 1815), while the species richness and abundance of lotic species (such as *Calopteryx splendens* (Harris, 1782), *Onychogomphus forcipatus* (Linnaeus, 1758), *Orthetrum coerulescens* (Fabricius, 1798)) decreased in the second year of the study. Adult Odonata generally have very good flight abilities, which allow them to travel among different waterbodies and over long distances [67,68]. However, lentic habitats are less predictable spatially and temporally than lotic habitats, which means that lentic species have better (higher) dispersal abilities crucial for their long-term survival [68,69].

Although results regarding Odonata body shape and reproduction type life history traits were not significant, we observed some trends regarding the time scale of the study. More precisely, a higher proportion of Zygoptera found in 2020 (compared to 2023) could be related to aforementioned extreme climate conditions in 2022 and 2023. Zygoptera tend to be less tolerant to high and low temperatures compared to Anisoptera, even if they occur at the same latitudes or altitudes [70,71]. As some anthropogenically disturbed lakes had well-developed vegetation, a higher proportion of endophytic oviposition reproduction trait (i.e., in species laying eggs in the substrate and plant tissue) (such as *Ischnura elegans*, *Ischnura pumilio* (Charpentier, 1825), *Platycnemis pennipes*) [51] was detected at such habitats in 2020 compared to 2023, most likely due to the higher abundance of Zygoptera in the first year of the study. Most Zygoptera have endophytic oviposition, and require substrate, such as aquatic macrophytes, to lay their eggs in [51,67].

### 4.2. Implications for Conservation

In this study, 13% of European and 28% of Croatian Odonata species were recorded in the studied urban wetland landscape [30,48]. Such species richness could be considered as moderately high [72]. However, this number is expected to rise with increasing sampling effort, because although adult Odonata are considered good indicators of habitat selection, their presence in a particular habitat is not a definitive indication of the species’ reproductive success there [56,67,72]. As suggested in previous studies, it is optimal to also examine nymphs and exuviae for the most accurate Odonata species list [34,73], which is highly recommended for future studies. Such addition would provide data about the species that complete their life cycles (i.e., are core residents) in the studied urban wetland. Moreover, further monitoring might be of use to determine how Odonata assemblages change over time, considering both natural and artificial drivers. Finally, the study focused on adults should also be conducted in spring as, in this way, additional species belonging to spring fauna could also be recorded. For instance, a previous study in this area reported *Brachytron pratense* (Müller, 1764) and *Epitheca bimaculata* (Charpentier, 1825) [39], which were not documented within the current study. Since there is sometimes a possibility that flying adults are double-counted (e.g., in case of high Odonata abundances), the mark and recapture method could be applied when studying adult Odonata.

As expected, *Platycnemis pennipes* and *Ischnura elegans* were the most common species at both anthropogenically disturbed and natural oxbow lakes, as they occur in a wide range of freshwater habitats [74]. Both species prefer to inhabit slow-flowing waters but are also frequently found in well-oxygenated stagnant waters with well-developed aquatic vegetation, such as oxbows and marshes or various man-made habitats (gravel pits, fishponds, canals) [57,74]. Although the values of the Dragonfly Biotic Index were comparable between the two habitat types, at natural oxbow lakes, some species of conservation concern were recorded: *Aeshna isoceles* and *Lestes sponsa* [48], which is consistent with the importance of natural habitat structure, with well-developed aquatic and riparian vegetation and a variety of microhabitats for rare Odonata [58].

## 5. Conclusions

Our results suggest that semi-natural urban wetlands have great potential to function as good habitats for Odonata. As these insects are generally evolutionary well-adapted to warmer conditions, and the majority of species recorded within this study are eurytherm generalists, able to tolerate wide temperature range in their habitats, this could indicate their tolerance to the UHI effects in urban waterbodies. Although most of taxonomic and functional assemblage metrics were comparable between the anthropogenically disturbed and natural oxbow lakes, species composition, but also abundance and some aspects of functional diversity (e.g., species preference for lateral habitat connectivity and their dispersal capacity), were different at the two habitat types, with species of conservation concern present only at the natural oxbow lakes. In addition, the observed significant differences in Odonata abundance with respect to the time scale of the study, indicate the negative impact of extreme climatic events on Odonata in urban habitats. The importance of habitat heterogeneity in terms of diverse aquatic macrophyte structure (presence of submerged, emergent and floating vegetation) and lateral connectivity must be considered when designing landscape management plans in urban areas to ensure the most suitable habitat conditions for local Odonata species.

## Figures and Tables

**Table 1:** Physico-chemical water properties measured in the anthropogenically disturbed and natural oxbow lakes in the Savica urban lakescape in the city of Zagreb, Croatia. The results of the generalized linear mixed models (GLMMs) show the differences in physico-chemical water properties between the habitats. Statistically significant effects and pairwise contrasts determined by the *post-hoc* least significant difference (LSD) test (*p* < 0.05) are in bold. Legend: SD—standard deviation, F—F-statistic, *p*—*p*-significance value, d.f.—degrees of freedom.

Physico-Chemical Water Parameter	Anthropogenically Disturbed Lake	Natural Oxbow Lake	F	*p*	d.f.
	Mean ± SD	Mean ± SD			
Water temperature (°C)	29.79 ± 1.87	27.40 ± 2.66	0.683	0.413	1
Oxygen saturation (%)	124.94 ± 16.13	97.24 ± 22.99	**4.566**	**0.038**	1
pH	7.81 ± 0.12	7.65 ± 0.27	0.976	0.329	1
Conductivity (µS/cm)	344 ± 19	315 ± 16	0.757	0.389	1
Water hardness (mg CaCO_3_/L)	188.82 ± 11.13	198.58 ± 10.48	2.009	0.164	1
Chemical oxygen demand (mg O_2_/L)	5.06 ± 0.90	4.14 ± 0.41	0.188	0.667	1
Nitrate concentration (mg N/L)	0.379 ± 0.122	0.086 ± 0.031	1.227	0.274	1
Nitrite concentration (mg N/L)	0.014 ± 0.002	0.005 ± 0.0003	**941.837**	***p* < 0.001**	1

**Table 2:** Abundance of Odonata species (shown as mean number of individuals) recorded during both years (2020 and 2023) of the study at anthropogenically disturbed and natural oxbow lakes in the Savica urban lakescape in the city of Zagreb, Croatia.

Species	Anthropogenically Disturbed Lakes	Natural Oxbow Lakes
*Calopteryx splendens* (Harris, 1782)	0.2	0.2
*Lestes sponsa* (Hansemann, 1823)		0.1
*Platycnemis pennipes* (Pallas, 1771)	23.1	26.5
*Ischnura elegans* (Vander Linden, 1820)	28.3	34.3
*Ischnura pumilio* (Charpentier, 1825)	0.7	0.1
*Erythromma viridulum* (Charpentier, 1840)	0.3	0.7
*Cordulia aenea* (Linnaeus, 1758)		0.1
*Onychogomphus forcipatus* (Linnaeus, 1758)		0.1
*Aeshna affinis* (Vander Linden, 1820)		0.1
*Aeshna isoceles* (Müller, 1767)		0.1
*Anax imperator* (Leach, 1815)	0.1	0.4
*Crocothemis erythraea* (Brullé, 1832)	0.5	1.1
*Libellula depressa* (Linnaeus, 1758)		0.2
*Orthetrum albistylum* (Selys, 1848)	1.4	1.0
*Orthetrum brunneum* (Fonscolombe, 1837)	0.2	0.3
*Orthetrum cancellatum* (Linnaeus, 1758)	0.3	0.2
*Orthetrum coerulescens* (Fabricius, 1798)	0.8	0.7
*Sympetrum sanguineum* (Müller, 1764)	0.6	0.7
*Sympetrum striolatum* (Charpentier, 1840)	0.2	0.8
Species richness (S)	13.0	19.0
Sum of all species abundances (mean, N)	56.5	67.4

**Table 3:** Results of SIMPER analysis based on Odonata assemblages from anthropogenically disturbed and natural oxbow lakes in the Savica urban lakescape in the city of Zagreb, Croatia. SIMPER analysis was based on the log-transformed species data and preformed using the Bray–Curtis similarity matrix.

**Group: anthropogenically disturbed lakes**		
Average similarity: 54.29		
**Species**	**Mean abundance** **per replicate**	**Similarity contribution** **within group (%)**
*Ischnura elegans*	2.97	56.66
*Platycnemis pennipes*	2.46	33.87
**Group: natural oxbow lakes**		
Average similarity: 55.14		
**Species**	**Mean abundance** **per replicate**	**Similarity contribution** **within group (%)**
*Ischnura elegans*	3.18	52.69
*Platycnemis pennipes*	2.72	38.28

**Table 4:** Results of the generalized linear mixed models (full models) (GLMMs) showing the differences in Odonata taxonomic assemblage and Dragonfly Biotic Index between anthropogenically disturbed and natural oxbow lakes in the Savica urban lakescape in the city of Zagreb, Croatia. Statistically significant effects resulting from the *post*-*hoc* least significant difference test (*p* < 0.05) are shown in bold. Legend: F—F statistic, d.f.—degrees of freedom, *p*—*p*-significance value, *t*—*t* test (the estimate divided by its standard error), D—disturbed habitat type, N—natural habitat type.

Taxonomic Parameter	Year	Habitat Type Pairwise Contrast	F	*p*	d.f.1	d.f.2	*t*	*p*
Species richness (S)	2020	D-N	0.259	0.853	3	26	0.000	1.000
	2023	D-N			3	26	0.913	0.424
	2020–2023	D-D			3	26	0.446	0.675
	2020–2023	N-N			3	26	0.220	0.834
Abundance (N)	2020	D-N	**3.317**	**0.035**	3	26	0.417	0.642
	2023	D-N			3	26	0.464	0.647
	2020–2023	D-D			3	26	1.382	0.179
	2020–2023	N-N			3	26	**2.041**	**0.050**
Simpson diversity (1 − λ)	2020	D-N	**4.563**	**0.047**	3	24	0.018	0.987
	2023	D-N			3	24	0.286	0.803
	2020–2023	D-D			3	24	2.344	0.108
	2020–2023	N-N			3	24	2.426	0.095
Shannon diversity (H′)	2020	D-N	0.693	0.577	3	26	0.536	0.606
	2023	D-N			3	26	0.486	0.639
	2020–2023	D-D			3	24	1.470	0.155
	2020–2023	N-N			3	24	0.310	0.155
Dragonfly Biotic Index (DBI)	2020	D-N	**4.385**	**0.045**	3	26	0.903	0.392
	2023	D-N			3	26	1.911	0.124
	2020–2023	D-D			3	26	0.970	0.341
	2020–2023	N-N			3	26	1.6699	0.376

**Table 5:** Results of the generalized linear mixed models (full models) (GLMMs) showing the differences in community weighted means (CWMs) of Odonata functional traits between anthropogenically disturbed and natural oxbow lakes in the Savica urban lakescape in the city of Zagreb, Croatia. Statistically significant effects resulting from the *post-hoc* least significant difference test (*p* < 0.05) are shown in bold. Legend: F—F statistic, d.f.—degrees of freedom, *p*—*p*-significance value, *t*—*t* test (the estimate divided by its standard error), D—disturbed habitat type, N—natural habitat type.

Functional Trait Group	Functional Trait	Year	Habitat Type Pairwise Contrast	F	*p*	d.f.1	d.f.2	*t*	*p*
Functional Diversity (RaoQ)		2020	D–N	0.927	0.422	3	26	1.424	0.116
		2023	D–N			3	26	0.863	0.396
		2020–2023	D–D			3	26	1.078	0.291
		2020–2023	N–N			3	26	1.100	0.281
Body shape	Anisoptera	2020	D-N	0.618	0.610	3	22	0.072	0.943
		2023	D-N			3	22	0.771	0.449
		2020–2023	D-D			3	22	1.116	0.276
		2020–2023	N-N			3	22	0.497	0.624
	Zygoptera	2020	D-N	1.587	0.218	3	25	0.445	0.660
		2023	D-N			3	25	0.680	0.503
		2020–2023	D-D			3	25	**2.005**	**0.050**
		2020–2023	N-N			3	25	0.596	0.556
Lateral connectivity preference	eupotamon	2020	D-N	**7.736**	**<0.001**	3	26	**2.117**	**0.044**
		2023	D-N			3	26	0.990	0.331
		2020–2023	D-D			3	26	**5.044**	**<0.001**
		2020–2023	N-N			3	26	**2.862**	**0.008**
	parapotamon	2020	D-N	**5.595**	**0.003**	3	26	1.539	0.136
		2023	D-N			3	26	1.095	0.284
		2020–2023	D-D			3	26	**2.088**	**0.047**
		2020–2023	N-N			3	26	**3.173**	**0.004**
	plesiopotamon (including lakes)	2020	D-N	**5.331**	**0.005**	3	26	0.757	0.456
		2023	D-N			3	26	0.244	0.809
		2020–2023	D-D			3	26	**3.197**	**0.004**
		2020–2023	N-N			3	26	**1.972**	**0.050**
	palaeopotamon (including pools, ponds)	2020	D-N	**16.677**	**<0.001**	3	26	**2.013**	**0.050**
		2023	D-N			3	26	0.005	0.996
		2020–2023	D-D			3	26	**5.136**	**<0.001**
		2020–2023	N-N			3	26	**3.486**	**0.002**
	temporary water bodies	2020	D-N	2.282	0.104	3	25	1.031	0.313
		2023	D-N			3	25	1.722	0.097
		2020–2023	D-D			3	25	0.736	0.469
		2020–2023	N-N			3	25	1.574	0.128
Current preference	limnophilous	2020	D-N	**6.732**	**0.002**	3	21	**2.008**	**0.050**
		2023	D-N			3	21	0.815	0.424
		2020–2023	D-D			3	21	**3.059**	**0.006**
		2020–2023	N-N			3	21	1.827	0.082
	limno- to rheophilous	2020	D-N	1.827	0.167	3	26	1.422	0.167
		2023	D-N			3	26	0.467	0.644
		2020–2023	D-D			3	26	1.150	0.143
		2020–2023	N-N			3	26	0.426	0.673
	rheo- to limnophilous	2020	D-N	0.000	1.000	3	9	0.000	1.000
		2023	D-N			3	9	0.000	1.000
		2020–2023	D-D			3	9	0.000	1.000
		2020–2023	N-N			3	9	0.000	1.000
	rheophilous	2020	D-N		0.000	-	-	-	-
		2023	D-N			-	-	-	-
		2020–2023	D-D			-	-	-	-
		2020–2023	N-N			-	-	-	-
Dispersal capacity	high	2020	D-N	**4.841**	**0.009**	3	25	1.244	0.225
		2023	D-N			3	25	0.149	0.882
		2020–2023	D-D			3	25	**3.357**	**0.003**
		2020–2023	N-N			3	25	**2.174**	**0.039**
	medium	2020	D-N	**4.466**	**0.013**	3	24	0.753	0.459
		2023	D-N			3	24	0.385	0.703
		2020–2023	D-D			3	24	**2.426**	**0.023**
		2020–2023	N-N			3	24	**2.359**	**0.027**
Reproduction	eggs attached to the substrate	2020	D-N	0.000	1.000	2	8	0.001	0.999
		2023	D-N			2	8	-	-
		2020–2023	D-D			2	8	-	-
		2020–2023	N-N			2	8	0.000	1.000
	eggs laid in the substrate	2020	D-N	1.744	0.183	3	26	0.310	0.759
		2023	D-N			3	26	1.110	0.277
		2020–2023	D-D			3	26	**2.102**	**0.045**
		2020–2023	N-N			3	26	0.635	0.531
	eggs not attached to or in the substrate	2020	D-N	**3.180**	**0.045**	3	21	0.088	0.930
		2023	D-N			3	21	0.509	0.616
		2020–2023	D-D			3	21	1.860	0.077
		2020–2023	N-N			3	21	1.927	0.068
	eggs laid in open water	2020	D-N	0.261	0.853	3	22	0.573	0.572
		2023	D-N			3	22	0.550	0.588
		2020–2023	D-D			3	22	0.266	0.793
		2020–2023	N-N			3	22	0.342	0.736
	eggs laid inside plant tissue	2020	D-N	1.744	0.183	3	26	0.326	0.747
		2023	D-N			3	26	1.104	0.280
		2020–2023	D-D			3	26	**2.102**	**0.045**
		2020–2023	N-N			3	26	0.635	0.531
	eggs laid onto plant material	2020	D-N	0.590	0.629	3	20	0.546	0.591
		2023	D-N			3	20	0.580	0.568
		2020–2023	D-D			3	20	0.379	0.709
		2020–2023	N-N			3	20	0.812	0.426
	eggs on exposed soil or rock	2020	D-N	0.214	0.812	2	7	0.488	0.641
		2023	D-N			-	-	-	-
		2020–2023	D-D			-	-	-	-
		2020–2023	N-N			2	7	0.318	0.760

## Data Availability

Data are available from the corresponding author upon request.
